# Exploring the Coinfection and Genetic Diversity of Multiple Tick-Borne Pathogens in Livestock Population of Punjab, Pakistan

**DOI:** 10.1155/2024/9958535

**Published:** 2024-01-29

**Authors:** Sabir Hussain, Abrar Hussain, Muhammad Umair Aziz, Baolin Song, Jehan Zeb, Sara Moutailler, Angélique Foucault-Simonin, Rebecca L. Smith, Alejandro Cabezas-Cruz, David George, Olivier Sparagano

**Affiliations:** ^1^Department of Infectious Diseases and Public Health, Jockey Club College of Veterinary Medicine and Life Sciences, City University of Hong Kong, Kowloon, Hong Kong SAR, China; ^2^School of Biological, Environmental, and Earth Sciences, University of Southern Mississippi, Hattiesburg, MS 39406, USA; ^3^Department of Pathobiology, College of Veterinary Medicine, Carle-Illinois College of Medicine, Department of Biomedical and Translational Sciences, University of Illinois, Urbana-Champaign 61802, USA; ^4^Anses, INRAE, Ecole Nationale Vétérinaire d'Alfort, UMR BIPAR, Laboratoire De Santé Animale, Maisons-Alfort F-94700, France; ^5^School of Natural and Environmental Sciences, Newcastle University, Newcastle Upon Tyne NE1 7RU, UK; ^6^Agricultural Sciences and Practice, Royal Agricultural University, Cirencester GL7 6JS, UK

## Abstract

Tick-borne diseases affecting domestic animals and humans have increased globally in recent years. Pakistan, in particular, faces a significant economic threat from ticks, where two specific species, *Rhipicephalus microplus* and *Hyalomma anatolicum*, act as vectors for various pathogens such as piroplasma, *Anaplasma*, *Ehrlichia*, and *Rickettsia* that pose a significant burden on livestock production in the country. To better understand the risk that tick-borne pathogens (TBPs) pose to livestock in Pakistan, we conducted a cross-sectional study of the occurrence, diversity, and coinfection of these pathogens in small and large ruminants owned by small farms as well as in ticks collected from these animals. We collected blood samples from 224 cattle, 224 buffalo, 69 goats, and 56 sheep, gathered from 112 farms located in seven districts of Punjab, one of Pakistan's largest province. In addition, we collected a total of 476 ticks attached to these animals. Based on the identification of tick species through morphology and sequence analysis of the 16S rRNA and cytochrome c oxidase subunit 1 (cox1) gene, we confirmed that the most commonly collected tick species were *Rh. microplus* (38.65% of all individuals), *H. anatolicum* (31.93%), and *Rh. decoloratus* (8.40%). Notable pathogens detected in the collected ticks included *Theileria annulata* (18.4% prevalence), *Anaplasma ovis* (15.79%), *A. centrale* (13.16%), and *Rickettsia slovaca* (13.16%). In blood samples, the most frequently detected pathogens were *T. annulata* (*n* = 8), *Babesia bovis* (*n* = 7), *A. centrale* (*n* = 6), and *B. bigemina* (*n* = 5). In some cases, both cattle and buffaloes were found to be coinfected with *B. bovis*, *T. annulata*, and *A. centrale*. These findings provide valuable insights into the circulation of TBPs in livestock and highlight the need for further research on the epidemiological risk that these pathogens pose to ruminants in Pakistan.

## 1. Introduction

Vector-borne diseases (VBDs), including tick-borne diseases (TBDs), are increasing globally, putting over half of the world's human population at risk and resulting in more than 1 million deaths annually [1]. The spatial distribution of TBDs is a growing public health concern due to climate change worldwide [2]. Ticks are the primary vectors for veterinary vector-borne pathogens (VBPs) and the second most significant vectors for human VBPs, following mosquitoes [1, 3]. Ticks are blood-sucking arachnid ectoparasites that infest various animals, including humans [2].

Ticks are classified into three major families: Ixodidae (949 known species), Argasidae (200 species), and Nuttalliellidae (one species) [4]. As arthropod vectors, ticks transmit a wide range of pathogens that affect domestic ruminants, leading to annual economic losses of US $13.9–$18.7 billion worldwide [4]. Ticks are also responsible for zoonotic diseases such as babesiosis, ehrlichiosis, Lyme disease, anaplasmosis, Rocky Mountain spotted fever, tick-borne relapsing fever, and tularemia [5, 6]. These TBDs impose economic restrictions on the global livestock sector, particularly in tropical and subtropical countries. Piroplasmosis (caused by protozoan parasites of the *Theileria* and *Babesia* genera), anaplasmosis (caused by *Anaplasma* species), and rickettsioses (caused by *Rickettsia* species) have a devastating impact on the livestock industry, with piroplasmosis and anaplasmosis being the most reported TBDs in ruminants [3, 7–9].

Livestock plays a vital role in the economy of Pakistan, particularly in rural areas [10]. The country has a substantial population of cattle (48 million), buffaloes (40 million), goats (78.2 million), and sheep (30.9 million), reared primarily by rural families and smallholders [11]. Numerous studies from Pakistan have reported that over 80% of the bovine population is infested with ticks, mainly from species of *Hyalomma* and *Rhipicephalus* [10, 12–14] which are known vectors for babesiosis, theileriosis, and anaplasmosis in ruminants and Crimean–Congo hemorrhagic fever (CCHF) in humans. A recent study identified as many as 30 tick species belonging to seven genera infesting large ruminants in Pakistan, alongside 40 species belonging to seven genera infesting small ruminants in the same study area [15]. This work found *Rhipicephalus microplus*, *Rhipicephalus annulatus*, and *Hyalomma anatolicum* to be the most common species infesting large ruminants, whereas on small ruminants, *H. anatolicum*, *Hyalomma dromedarii*, *Rh. microplus*, and *Rhipicephalus sanguineus sensu lato* (s.l.) were the most prevalent [15].

Coinfections from TBDs pose a threat to human and animal health worldwide, particularly in Pakistan. Coinfections with multiple TBPs can influence disease severity, alter disease signs and symptoms, and complicate diagnosis and treatment [16]. However, the risks and implications of acquiring coinfections are not yet fully understood [17], as they depend on tick bite exposure and the infection status of ticks themselves [18]. The risk of exposure to more than one pathogen from a single bite of a coinfected tick depends on both the prevalence of coinfections in ticks and the prevalence of coinfections in the hosts on which the ticks feed. In Pakistan, only two studies have focused on coinfections of TBPs in ticks [19, 20], but both neglected to undertake simultaneous assessment of coinfection frequencies in both ticks and hosts [21, 22].

The current research aims to address this gap by using the conventional polymerase chain reaction (PCR) and microfluidic-based high-throughput method. This method utilizes a small volume of nucleic acid to perform parallel real-time PCRs on 48 by 48 or 96 by 96 well chips, enabling the processing of up to 2,304 or 9,216 individual reactions, respectively [13, 23]. Employing this method has allowed the current study to assess the impact of TBPs on livestock animals in Pakistan.

## 2. Materials and Methods

### 2.1. Ethics Statement

Ethical approval for this study was obtained from the Institutional Research Ethics Committee of the City University of Hong Kong (internal reference number A-0672) after getting an import License from the Hong Kong government to analyze the samples in Hong Kong. All procedures were conducted in compliance with applicable guidelines and regulations. Furthermore, the study adhered to the STROBE-Vet (Strengthening the Reporting of Observational Studies in Epidemiology) guidelines.

### 2.2. Study Design

**Figure 1:**
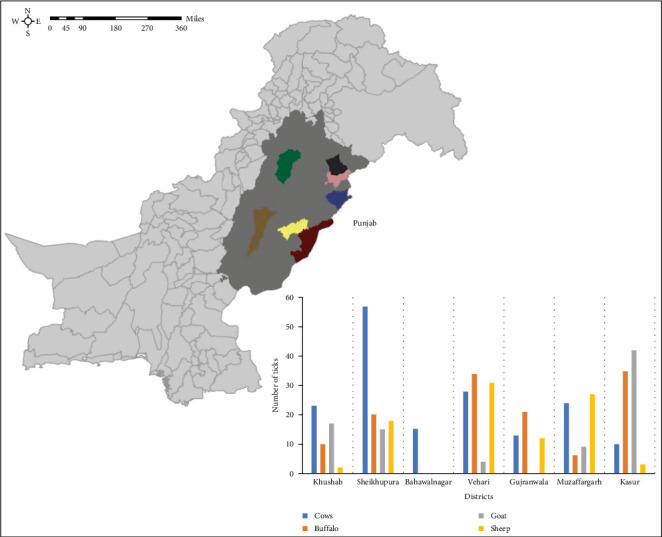
Locations and number of ticks collected from small and large ruminants in Punjab, Pakistan.

Pakistan, known for its agricultural landscape, comprises of four provinces, with Punjab being the largest. Punjab houses 36 districts, accommodating the highest animal and human populations in the country. Sampling was conducted between October 2020 and January 2021. The study focused on seven districts in Punjab, namely, Khushab, Bahawalnagar, Gujranwala, Kasur, Muzaffargarh, Sheikhupura, and Vehari (see Figure 1). These districts were chosen based on their high livestock populations and operational convenience. The selected farms housed herds ranged from 5 to 60 animals, mainly large ruminants such as cattle and buffaloes. A total of 16 farms were selected in each district to ensure representative sampling, and animals from the selected farms were chosen randomly using the random sampling tool provided by the Survey Toolbox software (Ausvet, The Australian Biosecurity Cooperative Research Centre for Emerging Infectious Disease, Australia). On each farm, four large ruminants (two cows and two buffaloes) were randomly selected for testing. In addition, if available, up to four small ruminants (two goats and two sheep) were also sampled. Ticks were also collected from at least two animals on each farm where tick infestation was observed. These ticks were then tested for TBPs as described in the later sections.

### 2.3. Blood Sample Collection and DNA Extraction

We collected 573 blood samples (8–10 ml per animal) from the jugular vein using disposable needles (Figure S1). For DNA extraction, we used the DNeasy Blood and Tissue Kit (Qiagen, Hilden, Germany) following the manufacturer's instructions. The only modification made was an increase in the incubation time from 10 to 30 min at 56°C for optimal results.

### 2.4. Tick Collection and Morphological Characterization

Ticks were collected from the animals during the winter season (October 2020–January 2021). Tick infestation of livestock is relatively low in Pakistan during winter, resulting in limited availability of ticks. Therefore, a uniform approach was followed, and two ticks were collected per animal. Once collected, the ticks were placed inside Eppendorf tubes and allowed to digest their blood meal for 36–48 hr. Subsequently, they were transferred to labeled 2 ml Eppendorf tubes containing 70% ethyl alcohol (Figure 1). Each hour tick specimen was carefully examined using a dissecting microscope (Olympus SZ40, Japan). The identification process involved using two complementary identification keys: Walker et al. [24] and online taxonomic keys included in Multikey 2.1 [10, 25]. In addition, reference was made to the original descriptions and redescriptions of relevant tick species.

### 2.5. Molecular Characterization of Ticks

From all seven study districts (Khushab, Bahawalnagar, Gujranwala, Muzaffargarh, Vehari, Kasur, and Sheikhupura), 476 ticks were collected. After morphological identification, each tick was washed three times in Milli-Q water after removing it from ethanol. The tick was then placed in 2 ml tubes and ground using a homogenizer (Precellys® 24 Touch, Item No. 36739) at 1,000 rpm for 2 min. DNA extraction was performed using the DNeasy Blood and Tissue Kit (Qiagen, Hilden, Germany) according to the recommended instructions. For five out of seven districts (Khushab, Bahawalnagar, Gujranwala, Muzaffargarh, and Vehari), 21 tick pools (consist of 276 ticks) were made based on the host within the district (i.e., all *H. anatolicum* collected from cattle in one district were in the same pool, whereas all other *H. anatolicum* collected from cattle in other districts were in separate pool). These 21 tick pools consisted of the following species: *Rh. microplus* (*n* = 7 pools), *H. anatolicum* (*n* = 7 pools), *Amblyomma variegatum* (*n* = 3 pools), *Ha. scupense* (*n* = 2 pools), *R. haemaphysaloides* (*n* = 1 pool), and *Rh. decoloratus* (*n* = 1 pool). Extracted DNA samples were identified from each pool by two mitochondrial genes (16S rRNA and Cox1) as previously published oligonucleotide primers (1–3) using conventional PCR in a thermal cycler (T100, BioRad). Primer sequences and all PCR conditions are given in Table S2. The PCR was carried out in a total volume of 25 *µ*l reaction mixture containing 1 *µ*l of each primer (10 pmol), 8 *µ*l of PCR grade water, and 12.5 *µ*l of DreamTaq Green PCR master mix with the cat. number 1082, and 2.5 *µ*l of genomic DNA. Known positive (*Rh. microplus*) and negative (milliQ H2O) controls were included in each run. PCR products were run on 2% agarose gel stained with Syber safe and visualized under a gel documentation system (BioRad California, USA). However, in the case of the remaining two districts, where livestock populations were highest (Kasur and Sheikhupura), individual ticks (*n* = 200) were molecularly identified through the process described above.

### 2.6. Molecular Characterization of Tick-Borne Pathogens in Blood and Ticks

All extracted DNA from 21 tick pools and blood samples DNA were further analyzed for the detection of the most common tick-borne pathogens (TBPs) circulating in small and large ruminants of all seven study districts of Punjab, Pakistan. PCR was carried out for 18S rRNA gene for the detection of piroplasm and sets of species-specific primer were used for the replication of 16S rRNA/*gltA* (*Anaplasma*/*Ehrlichia*/*Rickettsia*), 16S rRNA for *Borrelia* spp., and 18S rRNA for piroplasm. All the PCR reactions were carried out in a total volume of 25 *µ*l reaction mixture containing 1 *µ*l of each primer (10 pmol), 8 *µ*l of PCR grade water, 12.5 *µ*l of DreamTaq Green PCR Master Mix (cat. number 1082), and 2.5 *µ*l of genomic DNA. All the PCR conditions that were followed are detailed herein (Table S3). PCR products were run on 2% agarose gel stained with Syber safe and visualized under a gel documentation system (BioRad California, USA).

### 2.7. Sequencing and Phylogenetic Analysis of Ticks and Tick-Borne Pathogen

**Figure 2:**
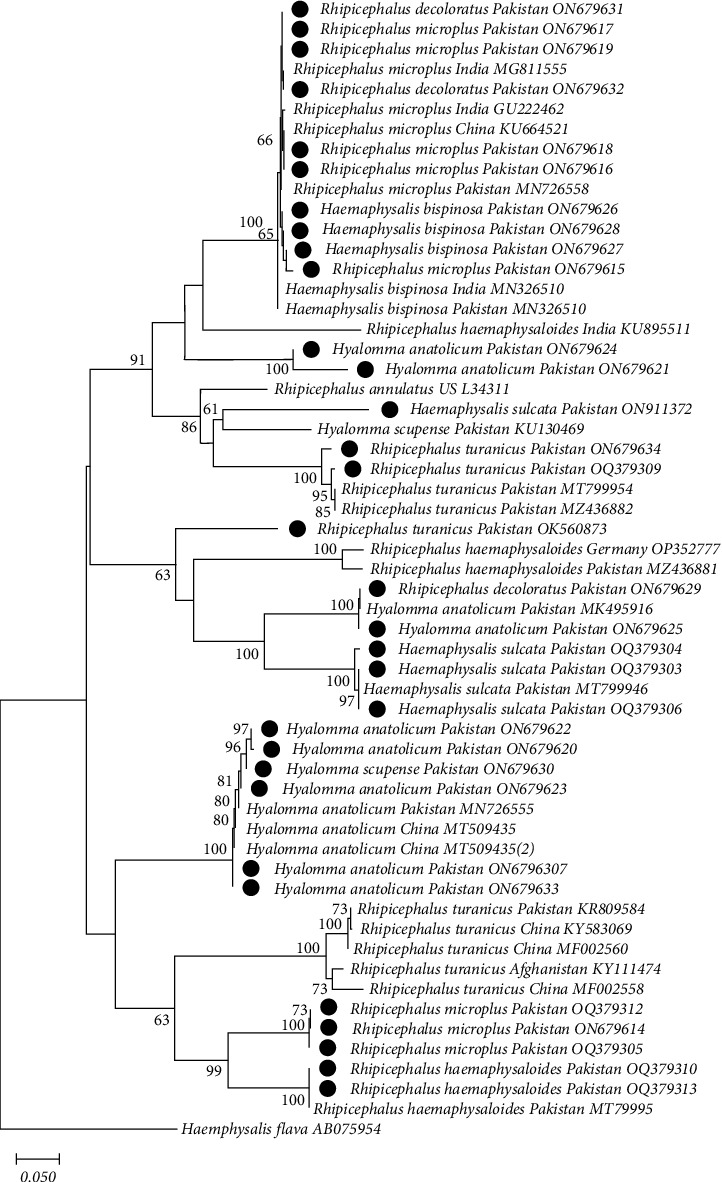
Genetic relationships of *Hyalomma* spp., *Haemaphysalis* spp., and *Rhipicephalus* spp. isolates from Punjab, Pakistan (black circles) with reference sequences selected from previous studies. The relationships were inferred based on the phylogenetic analyses of 16S rRNA gene partial sequence data using neighbor joining (NJ, this tree) and Bayesian inference (BI, not shown) methods, with *Haemaphysalis flava* used as the outgroup. The country of origin and GenBank accession numbers for each sequence are also provided. Node support values are indicated. The scale bar is also shown.

**Figure 3:**
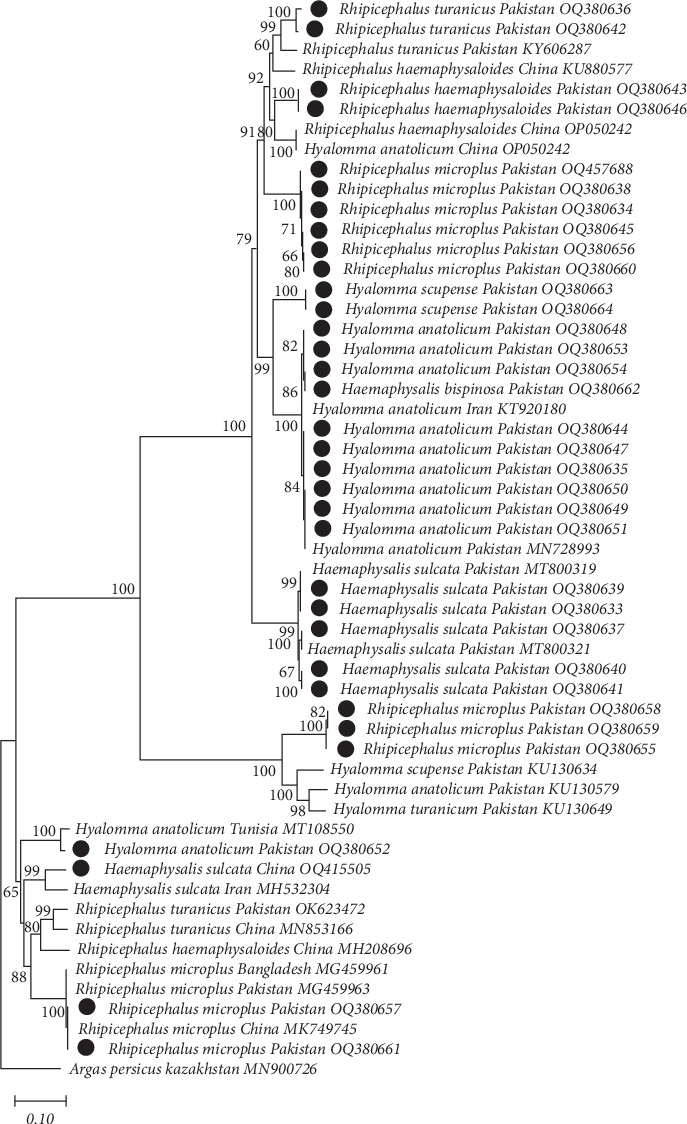
Genetic relationships of *Hyalomma spp*., *Haemaphysalis* spp., and *Rhipicephalus* spp. isolates from Punjab, Pakistan (black circles) with reference sequences selected from previous studies. The relationships were inferred based on the phylogenetic analyses of *cox1* gene partial sequence data using neighbor joining (NJ, this tree) and Bayesian inference (BI, not shown) methods, with *Haemaphysalis flava* used as the outgroup. The country of origin and GenBank accession numbers for each sequence are also provided. Node support values are indicated. The scale bar is also shown.

**Figure 4:**
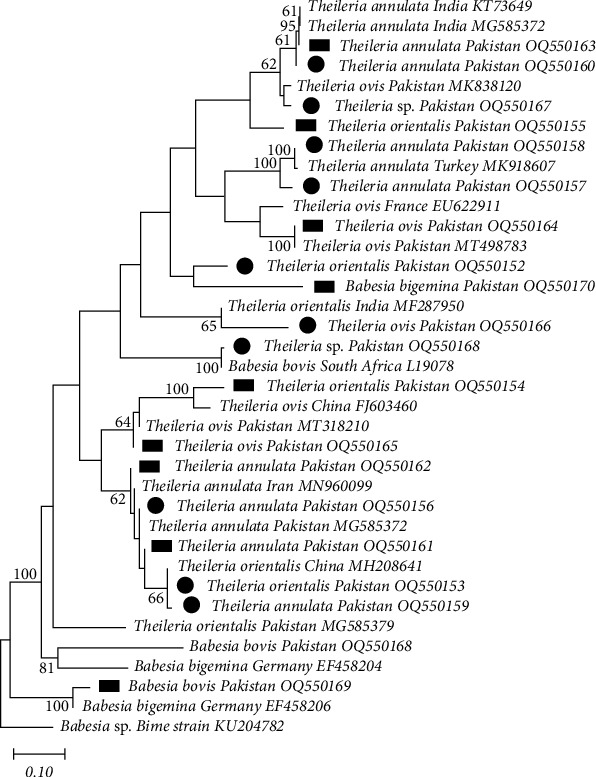
18S rRNA-based phylogenetic analysis of genotypes identified in *Babesia* and *Theileria* detected from a cross-sectional study of small and large ruminants in Punjab, Pakistan. Phylogenetic tree highlighting the position of genotypes from the present study (black circles indicate ticks and rectangles indicate blood samples) related to similar representative studies in the NCBI GenBank. The partial sequence of 18S rRNA gene was aligned and the phylogenetic tree was inferred in MEGA X using neighbor joining with the P-distance method with 1,000 bootstrap replications. Only bootstrap values higher than 50% are shown. *Babesia* sp. 18S RNA gene partial sequences was included as the outgroup. The scale bar is shown.

**Figure 5:**
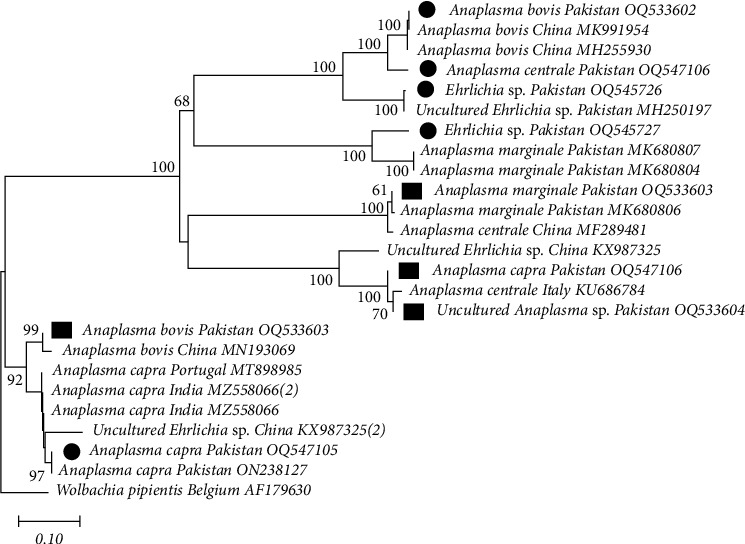
16S rRNA-based phylogenetic analysis of genotypes identified in *Anaplasma* and *Ehrlichia* spp. detected from a cross-sectional study of small and large ruminants in Punjab, Pakistan. Phylogenetic tree highlighting the position of genotypes from the present study (black circles indicate ticks and rectangles indicate blood samples) related to similar representative studies in the NCBI GenBank. The partial sequence of 16S rRNA gene was aligned and the phylogenetic tree was inferred in MEGA X using neighbor joining with the P-distance method with 1,000 bootstrap replications. Only bootstrap values higher than 50% are shown. *Wolbachia pipientis* 16S RNA (Belgium) gene partial sequences were included as the outgroup. The scale bar is shown.

**Figure 6:**
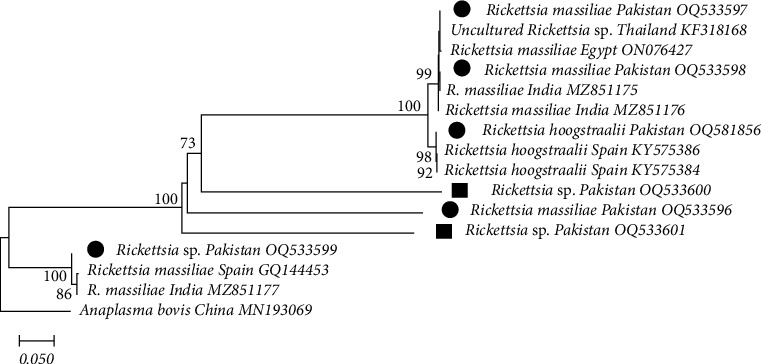
16S rRNA-based phylogenetic analysis of genotypes identified in *Rickettsia* spp. detected from a cross-sectional study of small and large ruminants in Punjab, Pakistan. Phylogenetic tree highlighting the position of genotypes from the present study (black circles indicate ticks and rectangles indicate blood samples) related to similar representative studies in the NCBI GenBank. The partial sequence of 16S rRNA gene was aligned and the phylogenetic tree was inferred in MEGA X using neighbor joining with the P-distance method with 1,000 bootstrap replications. Only bootstrap values higher than 50% are shown. *Anaplasma bovis* 16S RNA (China) gene partial sequences were included as the outgroup. The scale bar is shown.

**Figure 7:**
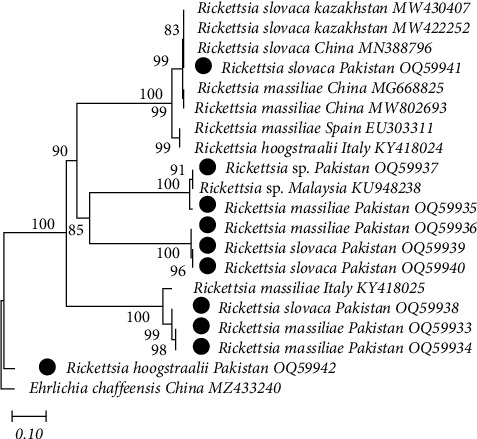
gltA-based phylogenetic analysis of *Rickettsia* spp. genotypes identified in a cross-sectional study of small and large ruminants in Punjab, Pakistan. Phylogenetic tree highlighting the position of genotypes from the present study (black circles indicate ticks and rectangles indicate blood samples) related to similar representative studies in the NCBI GenBank. The partial sequence of *gltA* gene was aligned and the phylogenetic tree was inferred in MEGA X using neighbor joining with the P-distance method with 1,000 bootstrap replications. Only bootstrap values higher than 50% are shown. *Babesia* sp.18S RNA gene partial sequences were included as the outgroup. The scale bar is shown.

All the purified PCR products were sent to BGI Tech Solutions Co. Ltd. (Hong Kong, SAR China) for sequencing. The nucleotide sequences obtained were edited and aligned using MUSCLE v.3.8.31 [5] within MEGA 11.0 using default settings [6]. These sequences were compared with the published sequences in the National Center for Biotechnology Information (NCBI) using BLASTn (https://blast.ncbi.nlm.nih.gov/Blast.cgi) to infer the specific identity of each tick and TBP. All the published sequences with query coverage of 99%–100% were downloaded and stored as separate data sets for further bioinformatics analyses. All the alignments were performed using MUSCLE, with default settings, and were trimmed to uniform lengths of 580 (Cox1) or 410 (16S rRNA) bp for the molecular identification of ticks and to uniform lengths of 490 (18S rRNA) bp for piroplasm; 330 (16S rRNA) bp for *Ehrlichia*, *Anaplasma*, *and Rickettsia*; and 310 (*gltA* for *Rickettsia*) bp for TBPs (from ticks and blood). The evolutionary models from all these sequence data were determined using the Akaike and the Bayesian information criteria tests in jModelTest v.3.7 [7]. Neighbor joining (NJ) trees were constructed using the Tamura–Nei distance method with 1,000 replicates for the molecular identification of both ticks (using 16S rRNA and Cox1) and TBPs (using 16S rRNA and 18S rRNA). In the analysis of 16S data, *Haemaphysalis flava*, Cox1 *Argas persicus* was used as an outgroup (Figures 2–7).

### 2.8. Microfluidic Real-Time PCR

We selected extracted ticks' DNA (Sheikhupura = 110 and Kasur = 90) and blood samples DNA (*n* = 74) from various tick species to conduct real-time microfluidic PCR. High-throughput microfluidic amplification was performed for major TBPs and potential endosymbionts using a 48.48 dynamics array in a BioMark^TM^ real-time PCR system (Standard Biotools, California, USA). These chips dispensed 48 samples and 48 PCR mixes into individual wells, followed by on-chip real-time PCR reactions in individual chambers and thermal cycling, resulting in 2,304 individual reactions. For more details regarding the development of this high-throughput tool based on real-time microfluidic PCRs (test of sensitivity, specificity, and controls used), see Michelet et al. [13]. Targeted microorganisms (and markers) were *Borrelia* spp. (23S), *Bo. burgdorferi* s.s. (*rpo*B), *Bo. garinii* (*rpo*B), *Bo. afzelii* (*flla*), *Bo. valaisiana* (*osp*A), *Bo. lusitaniae* (*rpo*B), *Bo. spielmanii* (*fla*), *Bo. bissettii* (*rpoB*), *Bo. miyamotoi* (*glp*Q), *Bo. mayonii* (*fla*), and *Bo. bavariensis* (*pyr*G); *Anaplasma* spp. (16S rRNA), *A. marginale* (msp1), *A. platys* (*gro*EL), *A. phagocytophilum* (msp2), *A. ovis* (msp4), *A. centrale* (*gro*EL), and *A. bovis* (*gro*EL); *Ehrlichia* spp. (16S), *E. canis* (*gltA*) and *Neoehrlichia mikurensis* (*gro*EL); *Rickettsia* spp. (*gltA*), *R. conorii* (ITS), *R. slovaca* (ITS), *R. massiliae* (ITS), *R. helvetica* (ITS), *R. aeschlimannii* (ITS), and *R. felis* (*orf*B); *Bartonella* spp. (*ssr*A), *Ba. henselae* (*pap31*); *Francisella* spp. (*tul4 and fop*A); *Coxiella* spp. (*IS1111 and icd*); *Babesia microti* (*CCTeta*), *B. canis* (18S), *B. ovis* (18S), *B. bovis* (CCTeta), *B. caballi* (*rap1*), *Babesia str. EU1* (*1*8S), *B. divergens* (*hsp70*), and *B. vulpes* (Cox1); *Theileria* spp. (18S); and *Hepatozoon* spp. (18S). Briefly, amplifications were performed using 6-carboxyfuorescein- (FAM-) and black hole quencher- (BHQ1-)labeled TaqMan probes with TaqMan Gene expression master mix according to the manufacturer's recommendations (Applied Biosystems, Massachusetts, USA) [13]. PCR cycling conditions comprised of a denaturation step at 95°C for 5 min followed by 45 cycles at 95°C for 10 s, 60°C for 15 s, and 40°C for 10 s. One negative control (water) was included per chip. Detection of ticks' 16S rRNA gene served as a positive control for the confirmation of DNA extraction. To assess PCR inhibitory molecules present in tick DNA samples, DNA from *Escherichia coli* (EDL933 strain) was added to each sample as an internal inhibition control, and primers and probes specific for the eae gene of *E. coli* were used. This PCR aimed to detect any of 47 microorganisms present in the ticks from these two districts. For a comprehensive list of the primers used in the microfluidic PCR, see Supplementary Materials.

### 2.9. Statistical Analysis

All analysis was run using R (open-source software version 4.2.1) [26]. The prevalence of each tick species and the prevalence of TBPs were calculated for both tick samples and blood samples.

## 3. Results

### 3.1. Tick Occurrence

**Figure 8:**
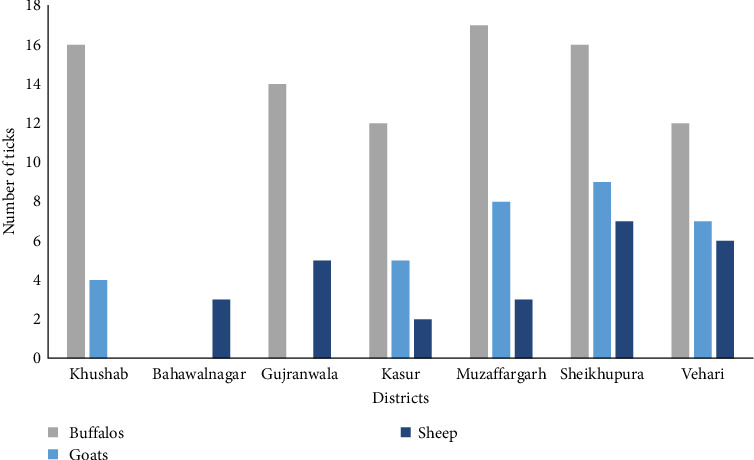
Number of animals in Punjab, Pakistan, from which ticks were collected.

Among the 112 farms surveyed, tick infestations were detected in 43 farms (38.4%; 95% CI 29.5–48.0), with variations observed across different subdistricts (see Table 1 and Figure 8). A total of 476 ticks were collected from seven districts: 97 from Vehari, 110 from Sheikhupura, 46 from Gujranwala, 90 from Kasur, 17 from Bahawalnagar, 50 from Khushab, and 66 from Muzaffargarh. To ensure accurate identification, all ticks underwent morphological and molecular analysis. Two specific genes, namely, 16S rRNA and *cox*1 gene were targeted for molecular identification. The most commonly identified tick species were *Rh. microplus* (*n* = 184), followed by *H. anatolicum* (*n* = 152), *Rh. decoloratus* (*n* = 40), and *Ha. bispinosa* (*n* = 30).

### 3.2. Molecular Characterization of Ticks

The obtained sequences represented various species of *Rhipicephalus*, *Haemaphysalis*, and *Hyalomma*. Detailed information about each tick species is provided in Tables S5 and S6, including their accession numbers after submission to GenBank. The submitted tick sequences showed similarity to other identical ticks of the same species reported worldwide, particularly in Pakistan (see Figures 2 and 3). Eight representative sequences of *Rh. microplus* from cows, buffaloes, sheep, and goats from each study district were deposited in the GenBank (16S: OQ379305, OQ379312, ON679614, ON679615, ON679616, ON679617, ON679618, and ON679619; Cox1: OQ380634, OQ380638, OQ380655, OQ380656, OQ380658, OQ380659, OQ380660, and OQ457688). These sequences were 100% identical to Pakistan (16S, MN726559), 99.70% identical to India (16S, MG11555 and GU222462), 99.30% similar to China (16S, KU664521), 99.50% similar to Pakistan (Cox1, MG459963), and a 99.20% similar to Bangladesh (Cox1, MG459961). Representative sequences of *H. anatolicum* from cows, buffaloes, sheep, and goats from each of these districts were deposited in the GenBank (16S: ON6796307, ON679620, ON679621, ON679622, ON679625, ON679633, and OQ380635; Cox1: OQ380644, OQ380647, OQ380648, OQOQ380653, OQ380654, OQ380649, OQ380650, OQ380651, and OQ380652). These sequences were 100% identical to Pakistan (16S, MK495916 and MN72655; Cox1: KU130579 and KU130649), 99.67% similar to China (16S, MT509435 and MT509434), 100% similar to Tunisia (Cox1, MT108550), and 99.20% similar to Iran (Cox1, KT920180). Representative sequences of *Rh. turanicus* (16S, ON679634 and OQ379309; Cox1, OQ380636 and OQ380642) were 100% identical to Pakistan (16S, KR809584; Cox, KY606287) and 99% similar to China (16S, KY583069 and MF002560; Cox1, MN853166) and Afghanistan (16S, KY111474). Similarly, *Rh. decoloratus* 16S rRNA (ON679629, ON679631, and ON679632) was 99.67% identical to India. Similarly, for *Rh. haemaphysaloides* (16S: OQ379310 and OQ379313; Cox1: OQ380643 and OQ380646), sequences were 100% identical to Pakistan (16S, MZ436881), 99.68% identical to India (16S, KU895511), 99.35% identical to Germany (16S, OP352777), 99.50% similar to Bangladesh (Cox1, MG459961), and 99.20% similar to China (Cox1, MH208696). Similarly, for *Haemaphysalis bispinosa* (16S: ON679626, ON679627, and ON679628; Cox1: OQ380662), samples were 99.68% identical to Pakistan (16S, ON679624) and 99.35% identical to India (16S, MN326510). Similarly, for 16S rRNA from *Ha. sulcata* (16S: OQ379303, OQ379304, OQ379306,0Q379309, and ON911372; Cox1: OQ380633, OQ380637, OQ380639, and OQ380640), samples were 100% identical to Pakistan (Cox1, MT800321). *Haemaphysalis flava* (16S, 1B075954) was used as an outgroup for 16S rRNA identification and *Argas persicu*s (Cox1, MN900726) was used for Cox1 identification (Tables S2 and S4; Figures 2 and 3).

**Figure 9:**
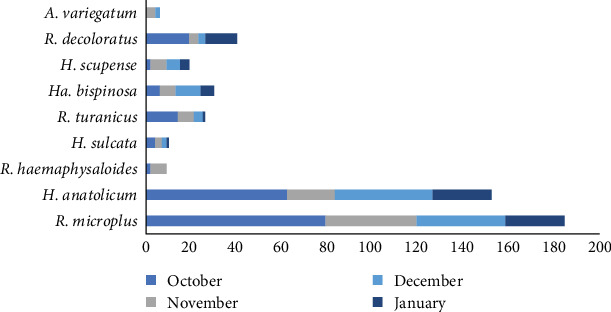
Variation in the number of different tick species collected during a 4 month cross-sectional study of small and large ruminants in Punjab, Pakistan.

Species-specific collection of ticks during different months of the collection period is shown in Figure 9.

### 3.3. Pathogen Detection in Ticks

Among the 21 pools of tick collected from the five districts (Khushab, Bahawalnagar, Gujranwala, Muzaffargarh, and Vehari), we identified a total of 14 different pathogen species through conventional PCR. The most commonly detected pathogens were as follows: *T. annulata* (*n* = 7), *A. ovis* (*n* = 6), *R. slovaca* (*n* = 5), *A. centrale* (*n* = 5), *R. massiliae* (*n* = 4), *A. marginale* (*n* = 4), *B. bigemina* (*n* = 4), *Theileria* sp. (*n* = 4), *T. ovis* (*n* = 3), *B. bovis* (*n* = 2), *A. capra* (*n* = 2), *Ehrlichia* sp. (*n* = 2), *R. hoogstraalii* (*n* = 2), *T. orientalis* (*n* = 1), and *A. bovis* (*n* = 1). Notably, no *Borrelia* species were detected in any of the ticks (Table 2).

The 200 ticks collected from the districts of Kasur (*n* = 90) and Sheikhupura (*n* = 110) were subjected to microfluidic PCR testing. The prevalence of any of the 47 pathogens was found to be 7.0% (*n* = 14) and 5.0% (*n* = 10) in the Kasur and Sheikhupura districts, respectively. The most prevalent microorganisms found in the ticks were as follows: *Anaplasma* sp. (*n* = 20, 10.0%) through 16S rRNA, *A. marginale* (*n* = 15, 7.5%) through msp1, *A. ovis* (*n* = 5, 2.5%), Apicomplexa (*n* = 4, 2.0%) through 18S rRNA, and *Theileria* sp. (*n* = 4, 2.0%) through 18S rRNA. In addition, *Ehrlichia* sp. (*n* = 1, 0.5%) was detected through 16S rRNA, *Rickettsia aeschlimannii* (*n* = 1, 0.5%) through ITS, and *Rickettsia* sp. (*n* = 1, 0.5%) through *gltA* gene. Single species infections with DNA from the above microorganisms were found in very few (*n* = 1, 0.5%) ticks. Among ticks, it was most common to find ‘double infections' with two species of microorganisms present (*n* = 21, 10.5%), followed by quadruple infections with four (*n* = 2, 1.0%) (Table S7).

### 3.4. Pathogens Detected in Blood Samples

Thirty out of 573 ruminant blood samples were tested positive (12 cattle, 12 buffalo, 3 sheep, and 3 goats), and total of 13 pathogens were identified. The most commonly detected pathogens were as follows: *T. annulata* (*n* = 8, 1.4%), *B. bovis* (*n* = 7, 1.2%), *A. centrale* (*n* = 6, 1.0%), *B. bigemina* (*n* = 5, 0.8%), *A. marginale* (*n* = 4, 0.7%), *T. ovis* (*n* = 3, 0.5%), *T. orientalis* (*n* = 3, 0.5%), *A. ovis* (*n* = 2, 0.3%), *R. slovaca* (*n* = 1, 0.2%), *A. capra* (*n* = 1, 0.2%), *Rickettsia* sp. (*n* = 1, 0.2%), *Ehrlichia* sp. (*n* = 1, 0.2%), and *A. bovis* (*n* = 1, 0.2%) (see Table 3).

Out of total 573 blood samples, 74 blood samples were collected from large ruminants in Kasur (*n* = 37) and Sheikhupura (*n* = 37) districts. These samples were screened for 47 microorganisms using high-throughput screening, with 15 out of 37 samples (40.54%) from Kasur and 29 out of 37 samples (78.37%) from Sheikhupura testing positive. The most prevalent microorganisms detected in the blood samples of bovines were *Anaplasma* sp. (*n* = 38, 51.35%) using 16S rRNA, followed by *A. marginale (n* = 19, 25.68%) using *msp1*, Apicomplexa (*n* = 18, 24.32%) using 18S rRNA, *A. ovis* (*n* = 17, 22.97%), and *Theileria* sp. (*n* = 16, 21.62%). In addition, *Anaplasma platys*, *Candidatus Anaplasma cinensis*, and *Candidatus Anaplasma camelii* were each detected in a single cattle blood sample (see Table S8).

### 3.5. Phylogenetic Analysis of TBPs from Tick and Blood Samples

To identify *Babesia* and *Theileria* species inside ticks and blood, nucleotide sequences of PCR products were analyzed and compared with the fragments of 18S rRNA sequences and previously published sequences obtained from GenBank. Through phylogenetic analysis, the 18S rRNA gene sequences were divided into two high homologous groups that were identified as different *Theileria* sp. from small and large ruminants in the seven study districts (ticks: OQ550152, OQ550153, OQ550156, OQ550157, OQ550158, OQ550159, OQ550160, OQ550166, OQ550167, OQ550168, and OQ845754; blood: OQ550154, OQ550155, OQ550162, OQ550163, OQ550164, OQ550165, OQ845756, and OQ845757) and *Babesia* species (blood: OQ550169 and OQ550170), by comparing with other sequences available in the GenBank database. The sequence similarity among these *Theileria* groups ranged from 99.20% to 99.65% and was very similar to *Theileria annulata* (KT73649, MG585372, MK918607, and MN96009), *T. orientalis* (MF287950, MH208641, and MG585379), and *T. ovis* (MG498783 and FJ603460). Similarly, sequence similarity of different *Babesia* species ranged from 99.30% to 99.85% and was very close to *B. bovis* (L19078 and OQ550168) and *B. bigemina* (EF550168, EF458204, and OQ845755). The *Babesia* sp. Bime strain (18S, KU20472) was used as an outgroup for the construction of this tree (Figure 4).

To identify *Anaplasma* and *Ehrlichia* species inside ticks and blood, nucleotide sequences of PCR products were analyzed and compared with the fragments of 16S rRNA sequences and previously published sequences obtained from GenBank. Through phylogenetic analysis, the 16S rRNA gene sequences were divided into two high homologous groups that were identified as four different *Anaplasma* sp. (ticks: OQ533602, OQ547106, OQ547105, OQ847800, OQ847801, OQ847802, and OQ847803; blood: OQ5533603, OQ547106, OQ533603, and OQ847804) and *Ehrlichia* sp. (ticks: OQ545726, and OQ54572) by comparing with other sequences available in the GenBank database. The sequence similarity among the four *Anaplasma* groups ranged from 99.35% to 99.75% and was very similar to *A. marginale* (MK680807 and MK680804), *A. bovis* (MK991954, MH255930, and MN193069), *A. centrale* (MF289481 and KU686784), and *A. capra* (MT898985 and MZ58066). Similarly, the sequence similarity among *Ehrlichia* groups ranged from 99.40% to 99.70% and was very homologous to the reported *Ehrlichia* sp. (KX987325). *Wolbachia pipientis* (AF1796300) was used as an outgroup for the construction of this phylogenetic tree (Figure 5).

**Figure 10:**
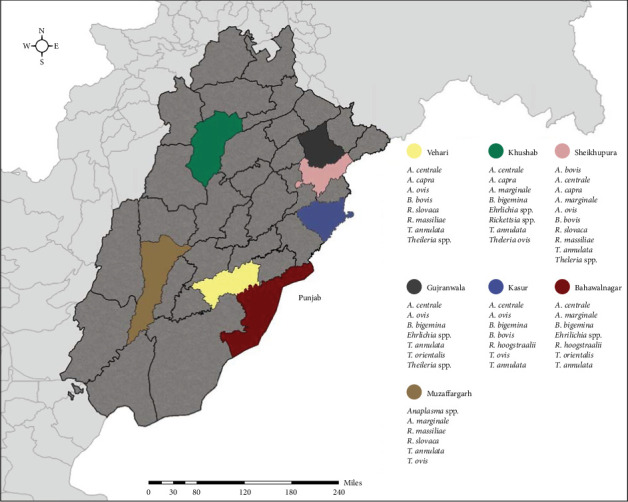
Tick-borne pathogens detected in ticks and blood from small and large ruminants of Punjab, Pakistan.

To identify *Rickettsia* species inside ticks and blood, nucleotide sequences of PCR products were analyzed and compared with the fragments of 16S rRNA sequences and previously published sequences obtained from GenBank. Through phylogenetic analysis, the 16S rRNA gene sequences that were identified as different *Rickettsia* sp. from small and large ruminants in the seven study districts (ticks: OQ533596, OQ533597, OQ533598, OQ533599, and OQ581856; blood: OQ533600 and OQ533601), by comparing with other sequences available in the GenBank database. The sequence similarity among these *Rickettsia* groups ranged from 99.25% to 99.55% and was very similar to *R. massiliae* (ON076427, MZ851175, MZ851176, GQ14453, and MZ851177), *Rickettsia* sp. (KF318168), and *R. hoogstraalii* (KY575386 and KY575384). The *Anaplasma bovis* (16S, MN193069) was used as an outgroup for the construction of this tree (Figure 6). Similarly, to identify *Rickettsia* species inside ticks and blood, nucleotide sequences of PCR products were analyzed and compared with the fragments of *gltA* gene sequences and previously published sequences obtained from GenBank. Using phylogenetic analysis, *gltA* gene sequences that were identified as different *Rickettsia* sp. from small and large ruminants in the seven study districts (ticks: OQ599533, OQ599534, OQ599535, OQ599536, OQ599537, OQ599538, OQ599539, OQ59940, and OQ599541; blood: OQ599542), by comparing with other sequences available in the GenBank database. The sequence similarity among these *Rickettsia* groups ranged from 99.45% to 99.75% and was very similar to *R. massiliae* (MG668825, MW802693, EU303311, and KY418025), *Rickettsia* sp. (OQ59937), *R. hoogstraalii* (KY418024), and *R. slovaca* (MW430407, MW422252, and MN388796). The *Ehrlichia chaffeensis (gltA*, MZ433240) was used as an outgroup for the construction of this tree (Table S9 and Figures 7 and 10).

### 3.6. Coinfection of TBPs in Small and Large Ruminants

Multiple pathogen associations were identified in the blood samples. In cattle from the Vehari district, coinfection of *B. bovis* and *T. annulata* was observed. In cattle from Sheikhupura, coinfection of *T. annulata* and *A. bovis* was found, and in Gujranwala, coinfection of *A. centrale*, *T. orientalis*, and *B. bigimina* was found. Buffalo from Gujranwala showed coinfection of *A. centrale* and *T. annulata*, whereas buffalo and cattle from the same district were coinfected with *A. centrale* and *B. bovis*. Buffalo from Bahawalnagar exhibited coinfection of *A. centrale* and *T. annulata*, and in Khushab, these animals were found to be coinfected with *B. bigemina* and *A. centrale*. Goats from Khushab were also coinfected with TBPs, in this case *T. ovis* and *A. capra* (Table 3).

In Sheikhupura, *Rh. microplus* collected from cattle showed coinfection of *A. marginale* and *T. annulata*, whereas *Rh. microplus* collected from sheep exhibited coinfection of *A. ovis*, *R. slovaca*, and *R. massiliae*. *H. anatolicum* collected from sheep were coinfected with *A. marginale* and *A. bovis*, and the same species collected from sheep also showed coinfection of *A. ovis*, *R. slovaca*, *R. massiliae*, and *A. centrale*. *Rhipicephalus turanicus* collected from buffalo in Gujranwala displayed coinfection of *Theileria* species, *A. centrale*, *R. slovaca*, and *R. massiliae*. In Kasur, *Rh. microplus* collected from goats were coinfected with *T. ovis*, whereas *B. bigemina* and *Rh. decoloratus* collected from buffalo exhibited coinfection with *A. centrale* and *B. bovis*. Finally, *Ha. sulcata* collected from sheep in Kasur showed coinfection of *A. ovis* and *R. hoogstraalii* (Table 2).

## 4. Discussion

### 4.1. Tick Distribution

This study represents the first investigation in Pakistan to collect ticks and blood samples from the same animals, providing valuable insights into the prevalence of pathogens and coinfections. Our findings indicate that the genus *Rhipicephalus* were the most commonly collected ticks from both small and large ruminants in Punjab, Pakistan, followed by *Hyalomma*, *Haemaphysalis*, and *Amblyomma*. This distribution pattern aligns with relative abundance reports from various regions in Pakistan [15, 20, 27–29]. Previous studies identified 30 tick species on cattle and buffalo as well as 40 tick species on sheep and goats in Pakistan [15]. However, most of these studies relied on morphological identification [13, 30–44], with only a few providing molecular data [10, 11, 45].

Our study is the first to molecularly characterize *Rhipicephalus decoloratus* in Pakistan, a tick species widely reported in neighboring countries such as India [46]. The presence of this tick species in Pakistan may be attributed to migratory birds, trade, or animal movement across the border. Moreover, our study marks the first recorded instance of *A. variegatum* in three districts of Punjab, Pakistan. Although this tick species has been previously reported in donkeys in Khyber Pakhtunkhwa and cattle in Balochistan, this is the first documentation of its presence in sheep, goats, and cattle.

Among the ticks we identified, 38% were *Rh. microplus*, which has the potential to act as a vector for various pathogens, including *B. bigemina*, *B. bovis* (bovine babesiosis), and *A. marginale* (anaplasmosis) [47]. In addition, our study identified *H. anatolicum*, a known vector for *T. annulata*, *T. lestoquardi*, *T. equi*, *B. caballi*, and the CCHF virus [48]. Other studies investigating ticks collected from ruminants in the region have reported similar relative abundances of these two tick species [10]. These two ticks are responsible for the transmission of most endemic TBDs in Pakistan's ruminant population, and the increasing incidence of these diseases can be attributed to rising temperatures and humidity levels, which create favorable conditions for tick proliferation [49]. Climate change and global warming significantly impact tick development and survival, as they are dependent on specific climate conditions for their various life stages [50]. Furthermore, the availability of hosts and vegetation also influences tick population dynamics [50]. Records of tick species in Pakistan from 1947 to 2021 indicate a continuous increase in tick populations over this period, primarily driven by the expansion of vector habitats resulting from irregular rainy seasons during the past two decades and the decline in vector predator populations [15].

### 4.2. Tick-Borne Pathogen Distribution

Our study revealed that the ticks sampled carried various TBPs, primarily species of *Anaplasma*, *Theileria*, and *Babesia*, which are responsible for causing a range of significant diseases. The widespread distribution of these pathogens among the ticks collected from our seven districts emphasizes the continuous threat they pose to the livelihoods of smallholder farmers in Pakistan.

Through conventional PCR, we determined that the prevalence of TBPs among the ruminant population in Punjab, Pakistan, was 5.23%. Another study conducted in selected areas of Pakistan, including Chakwal, Jhang, and Faisalabad districts, found a higher prevalence of major TBPs (babesiosis, theileriosis, and anaplasmosis) at 25.26% (144 out of 450) [51]. The lower prevalence observed in our study could be attributed to winter season sampling, where tick presence is typically higher in the summer when populations thrive in hot and humid weather conditions [52]. The current work also reported lower prevalence of other TBPs, potentially for the same reason. In our study, the prevalence of theileriosis was found to be 2.01% in large ruminants and 0.34% in small ruminants. In comparison, a study conducted in Lahore, Punjab, reported a higher prevalence of 11.2% in large ruminants [53, 54], whereas in the Malakand division, the prevalence was 3.44% in small ruminants and 6.21% in large ruminants [54]. For babesiosis, we reported a prevalence of 2.09% (with 12 positives out of 573 samples) in large ruminants, with higher prevalence (13.89%) again reported in other work across three districts in Punjab, Pakistan [55]. In this same study, the prevalence of babesiosis in small ruminants ranged from 15.5% to 48.50%. Conversely, we did not find any cases of babesiosis among the small ruminants sampled, possibly due to the smaller sample size for blood collection.

Regarding anaplasmosis, we found a prevalence of 1.91% in large ruminants and 0.52% in small ruminants, this once again being lower than figures presented in preceding research. In a previous study, the prevalence of anaplasmosis in small ruminants ranged from 13.5% to 51.52%, for example, and was 16.2% in large ruminants across Pakistan [56]. The prevalence of ehrlichiosis and rickettsiosis in the current study was found to be 0.17% in small ruminants and 0.17% in large ruminants, with work elsewhere similarly reporting higher prevalence rates of 9.38% in goats and 6.25% in sheep in Pakistan [57].

Our study also identified the presence of *R. hoogstraali* in ticks collected from sheep in the Kasur and Bahawalnagar districts in Punjab, Pakistan, confirmed through the *gltA* gene. Previous studies have also detected *R. hoogstraali* in *Ha. sulcata* (hard ticks) and *Carios capensis* (soft ticks) [58], and it is known to be widely prevalent in Europe and North America [59], and present in neighboring countries. In Iran, for example, *R. hoogstraali* has been detected in *Ha. montgomeryi* from sheep and goats, indicating the potential movement of this pathogen across the border, most likely through birds and animal migration. Using a phylogenetic tree based on the 18S RNA genomic region, we found that most of the *Theilera* species isolated in our study aligned with sequences from Pakistan (MK838120, MT498783, and MG585379). However, some species isolated in our study belonged to other clades on this tree, indicating genetic diversity of *Theilera* species in our samples and linkages to clades associated with India (MG585382 and MF287950), Iran (MN96099), Turkey (MK918607), China (FJ603460 and MH2086410), and France (EU622911). Similarly, many of the *Babesia* species isolated in our study also clustered with those found in small and large ruminants in Pakistan (OQ550168), but with other strains aligning to different clades on the tree. This finding suggests that *Babesia* species in Pakistan exhibit genetic diversity, with some similarities to *B. bovis* strains from South Africa (L19078) and *B. bovis* strains from Germany (EF4582040 and EF4582026), which is consistent with previous studies [26]. The diversity of *Babesia* and *Theilera* species in Pakistan may be due to their biological variability and recombination between different genotypes.

### 4.3. Coinfection of the Tick-Borne Pathogen; a New Direction

This study presents the first investigation of coinfections of TBPs in ticks and the blood of their hosts using conventional and microfluidic PCR techniques in Pakistan. With conventional PCR, we identified the most common coinfections as *B. bovis* and *T. annulata* (*n* = 3), *A. centrale* and *T. annulata* (*n* = 2), and *A. centrale* and *B. bovis* (*n* = 2). These major pathogens, frequently observed in Pakistan, are known to cause babesiosis, theileriosis, and anaplasmosis in animals. Our findings are consistent with these known pathogens and highlight their significant economic impact.

In the case of microfluidic PCR, coinfections of *Anaplasma platys*, *Candidatus Anaplasma cinensis*, and *Candidatus Anaplasma camelii* were detected in cattle from the Sheikhupura district. A previous study conducted in Morocco identified *H. anatolicum* as a suspected transmitting vector for *Ca. Anaplasma camelii* [60], suggesting that *H. anatolicum* may also contribute to the presence of *Ca. Anaplasma camelii* in the cattle population of our study. Similarly, in studies of camels in Iran, 15% (30/200) infection by *Candidatus Anaplasmac amelii*, respectively [61]. *Anaplasma platys* has been reported in humans, sheep, cattle, and cats, indicating potential cross-species transmission of this pathogen [62].

Furthermore, we found coinfection of *A. ovis*, *R. slovaca*, and *R. massiliae* in two tick species, *H. anatolicum* and *Rh. microplus*, collected from sheep in the Sheikhupura district. These findings suggest that *Rh. microplus* could be a competent vector for the transmission of these pathogens. Another study provided evidence of *Rh. microplus*' vector competence for the transmission of *A. marginale*, demonstrating transovarial transmission through controlled experiments [63]. However, it is important to note that our identification of pathogens in cattle blood and ticks from those cattle does not provide definitive proof of vector competence. Further research is needed to investigate the vector competence of various ticks, including *Rh. microplus* and *H. anatolicum*, to enhance our understanding of the epidemiological distribution and coinfection patterns of TBPs.

## 5. Conclusions

The current study, conducted on tick occurrence and pathogen detection in ruminants, yielded several significant findings. Tick infestations were observed in 37.5% (43 out of 112) of the surveyed farms, with variations across different subdistricts. The most commonly identified tick species were *Rh. microplus*, *H. anatolicum*, *Rh. decoloratus*, and *Ha. bispinosa* targeting the 16S rRNA and *cox1* genes. The obtained tick sequences exhibited similarity to ticks of the same species reported worldwide, particularly in Pakistan. In addition, 14 different pathogen species were detected in the collected ticks, including *T. annulata*, *A. ovis*, *R. slovaca*, *A. centrale*, *R. massiliae*, *A. marginale*, *B. bigemina*, *Theileria* sp., *T. ovis*, *B. bovis*, *A. capra*, *Ehrlichia* sp., *R. hoogstraalii*, *T. orientalis*, and *A. bovis*. Further analysis focused on pathogen detection in blood samples, where 13 pathogens were identified, including *T. annulata*, *B. bovis*, *A. centrale*, *B. bigemina*, *A. marginale*, *T. ovis*, *T. orientalis*, *A. ovis*, *R. slovaca*, *A. capra*, *Rickettsia* sp., *Ehrlichia* sp., and *A. bovis*. Phylogenetic analysis was performed using specific genetic markers, which allowed the construction of phylogenetic trees for different species, such as *Babesia*, *Theileria*, *Anaplasma*, *Ehrlichia*, and *Rickettsia*. Coinfection of multiple pathogens was observed in both small and large ruminants, highlighting the complexity of pathogen associations. These findings contribute to our understanding of the epidemiology and distribution of TBDs, which can aid in the development of effective control and prevention strategies in veterinary medicine.

## Figures and Tables

**Table 1:** Details of ticks collected from small and large ruminants in different districts of Punjab, Pakistan.

Districts	Farms with ticks	Host	Tick species	Adult male	Adult female	Nymph	Total
Vehari	8 (50%)	Cattle	*Rh. microplus*	7	21	0	28
		Sheep	*Rh. microplus*	13	17	1	31
		Buffalo	*H. anatolicum*	18	13	3	34
		Goat	*Rh. haemaphysaloides*	0	3	1	4

Sheikhupura	8 (50%)	Cattle	*Rh. microplus*	13	18	0	31
			*H. anatolicum*	9	17	0	26
		Sheep	*H. anatolicum*	0	8	1	9
			*Rh. microplus*	7	0	2	9
		Goat	*Ha. sulcata*	4	0	0	4
			*Rh. turanicus*	0	6	0	6
			*Rh. haemaphysaloides*	1	4	0	5
		Buffalo	*Rh. turanicus*	12	8	0	20

Gujranwala	5 (31%)	Cattle	*Rh. microplus*	12	1	0	13
		Sheep	*Rh. microplus*	8	2	1	11
			*H. anatolicum*	1	0	0	1
		Buffalo	*H. anatolicum*	5	16	0	21

Kasur	7 (44%)	Goat Goat	*Rh. microplus*	19	2	1	22
			*H. anatolicum*	1	11	0	12
			*Ha. sulcata*	0	1	2	3
			*Ha. bispinosa*	5	0	0	5
		Cattle	*Ha. bispinosa*	4	2	0	6
			*H. scupense*	3	1	0	4
		Buffalo	*Rh. decoloratus*	11	5	0	16
			*Ha. bispinosa*	0	18	1	19
		Sheep	*Ha. sulcata*	0	3	0	3

Bahawalnagar	5 (31%)	Cattle	*H. anatolicum*	12	2	1	15
		Sheep	*A. variegatum*	0	2	0	2

Khushab	2 (13%)	Cattle	*H. scupense*	1	7	1	9
			*A. variegatum*	0	2	0	2
			*H. anatolicum*	0	12	0	12
		Goat	*Rh. microplus*	11	6	0	17
		Buffalo	*Rh. microplus*	1	8	1	10

Muzaffargarh	7 (44%)	Cattle	*Rh. decoloratus*	15	9	0	24
		Buffalo	*H. scupense*	4	2	0	6
		Goat	*H. anatolicum*	5	2	0	7
			*A. variegatum*	0	2	0	2
		Sheep	*H. anatolicum*	7	7	1	15
			*Rh. microplus*	8	3	1	12

Total	42 (38%)			219	239	18	476

**Table 2:** Details of pathogens detected from ticks taken from small and large ruminants in different districts of Punjab, Pakistan, as identified using 18S rRNA, 16S rRNA, and *gltA* gene.

District	Host	Pathogen detected in ticks	Tick species
Vehari	Cattle	—	*H. anatolicum*
	Sheep	*A. ovis*, *Theileria* spp.	*Rh. microplus*
	Cattle	*B. bovis*, *T. annulata*, *A. centrale*	*Rh. microplus*
	Goat	*A. capra*, *A. centrale*, *R. slovaca*, *R. massiliae*, *Theileria* spp.	*Rh. haemaphysaloides*

Sheikhupura	Cattle	*A. marginale*, *T. annulata*	*Rh. microplus*
	Cattle	*A. marginale*, *A. bovis*	*H. anatolicum*
	Sheep	*A. ovis*, *R. slovaca*, *R. massiliae*, *A. centrale*	*H. anatolicum*
	Sheep	*A. ovis*, *R. slovaca*, *R. massiliae*	*Rh. microplus*
	Goat	—	*Rh. turanicus*
	Goat	—	*Rh. haemaphysaloides*
	Goat	*A. capra*	*Ha. sulcata*
	Buffalo	*Theleria* spp., *A. centrale*, *R. slovaca*, *R. massiliae*	*Rh. turanicus*

Gujranwala	Cattle	*A. centrale*, *T. orientalis*, *B. bigemina*	*Rh. microplus*
	Sheep		*Rh. microplus*
	Sheep	*A. ovis*, *Ehrlichia* spp., *Theileria* spp.	*H. anatolicum*
	Buffalo	*A. centrale*, *T. annulata*	*H. anatolicum*

Kasur	Goat	*T. ovis*, *B. bigemina*	*Rh. microplus*
	Goat	—	*H. anatolicum*
	Goat	—	*Ha. sulcata*
	Cattle	—	*Ha. bispinosa*
	Buffalo	*T. annulata*	*Ha. bispinosa*
	Goat	*B. bigemina*	*Ha. bispinosa*
	Cattle	*T. annulata*	*H. scupense*
	Buffalo	*A. centrale*, *B. bovis*	*Rh. decoloratus*
	Sheep	*A. ovis*, *R. hoogstraalii*	*Ha. sulcata*

Bahawalnagar	Buffalo	*A. centrale*, *T. annulata*	*H. anatolicum*
	Sheep	*R. hoogstraalii*	*A. variegatum*

Khushab	Cattle	—	*Ha. scupense*
	Cattle	*Ehrlichia* sp., *A. marginale*, *T. annulata*	*H. anatolicum*
	Buffalo	*B. bigemina*, *A. centrale*	*Rh. microplus*
	Goat	*Theleria ovis*, *A. capra*	*Rh. microplus*
	Cattle	—	*A. variegatum*

Muzaffargarh	Cattle	*A. marginale*	*Rh. decoloratus*
	Buffalo	*T. annulata*, *R. massiliae*	*Ha. scupense*
	Goat	—	*H. anatolicum*
	Sheep	*R. slovaca*, *T. annulata*	*H. anatolicum*
	Sheep	—	*Rh. microplus*
	Goat	*T. ovis*, uncultured *Anaplasma* sp.	*Rh. microplus*

**Table 3:** Details of pathogens detected from blood taken from small and large ruminants in different districts of Punjab, Pakistan, as identified using 18S rRNA, 16S rRNA, and *gltA* gene.

District	Host	Pathogens detected in blood samples
Vehari	Sheep	*A. ovis*
	Cattle	*B. bovis*, *T. annulata*
	Cattle	*B. bovis*, *T. annulata*

Kasur	Cattle	*A. centrale*, *B. bovis*
	Cattle	*T. annulata*
	Cattle	*T. annulata*
	Buffalo	*B. bigemina*
	Buffalo	*A. centrale*, *B. bovis*

Sheikhupura	Cattle	*A. marginale*
	Cattle	*T. annulata*, *A. bovis*
	Cattle	*B. bovis*, *T. annulata*
	Buffalo	*B. bovis*
	Goat	*A. ovis*

Gujranwala	Cattle	*A. centrale*, *T. orientalis*, *B. bigemina*
	Buffalo	*A. centrale*, *T. annulata*
	Sheep	*T. ovis*
	Buffalo	*T. orientalis*
	Buffalo	*T. annulata*
	Cattle	*T. annulata*
	Buffalo	*A. centrale*, *B. bovis*
	Cattle	*A. centrale*, *B. bovis*
	Buffalo	*B. bigemina*

Bahawalnagar	Buffalo	*A. centrale*, *T. annulata*
	Cattle	*A. marginale*
	Cattle	*T. orientalis*
	Buffalo	*B. bigemina*
	Goat	*Ehrlichia* spp.

Khushab	Goat	*T. ovis*, *A. capra*
	Buffalo	*B. bigemina*, *A. centrale*
	Cattle	*B. bigemina*
	Buffalo	*Rickettsia* spp.

Muzaffargarh	Cattle	*A. marginale*
	Sheep	*R. slovaca*
	Buffalo	*A. marginale*

## Data Availability

All data generated and analyzed during this study are included as supplementary information files.

## Additional Points

*Limitations*. As our study is based on convenience so there can be bias in reporting the tick's prevalence and infestation. There could be biased in reporting the prevalence of TBPs for five districts but for the districts with higher livestock populations, ticks were individually screened for TBPs.

## Supplementary Materials

Supplementary Materials Figure S1: blood collection details from small and large ruminants in Punjab, Pakistan. Table S1: numbers of blood samples obtained by district from small and large ruminants in Punjab, Pakistan. Table S2: primer details for molecular identification of ticks. Table S3: primer details for molecular identification of pathogens. Table S4: species and numbers of ticks collected by district and host, along with their accession numbers. Table S5: tick 16S rRNA query sequences similarities with GenBank subject sequences. Table S6: tick Cox1 query sequences similarities with GenBank subject sequences. Table S7: microorganisms detected through real-time microfluidic PCR in the individual tick species, collected from bovines in the districts of Kasur and Sheikhupura. Table S8: microorganisms detected through real-time microfluidic PCR in the blood samples of bovine populations in the study districts of Kasur and Sheikhupura. Table S9: tick-borne pathogens (TBPs) detected from ticks and blood using 16S rRNA, gltA, and 18S rRNA query sequence similarities with GenBank subject sequences.
