# Supplementary material for: Exploring the Coinfection and Genetic Diversity of Multiple Tick-Borne Pathogens in Livestock Population of Punjab, Pakistan
Source: Transbound Emerg Dis. 2024 Jan 29;2024:9958535. doi: 10.1155/2024/9958535 (PMC12016990; doi:10.1155/2024/9958535)
Supplement: Supplementary Materials — Figure S1: blood collection details from small and large ruminants in Punjab, Pakistan. Table S1: numbers of blood samples obtained by district from small and large ruminants in Punjab, Pakistan. Table S2: primer details for molecular identification of ticks. Table S3: primer details for molecular identification of pathogens. Table S4: species and numbers of ticks collected by district and host, along with their accession numbers. Table S5: tick 16S rRNA query sequences similarities with GenBank subject sequences. Table S6: tick Cox1 query sequences similarities with GenBank subject sequences. Table S7: microorganisms detected through real-time microfluidic PCR in the individual tick species, collected from bovines in the districts of Kasur and Sheikhupura. Table S8: microorganisms detected through real-time microfluidic PCR in the blood samples of bovine populations in the study districts of Kasur and Sheikhupura. Table S9: tick-borne pathogens (TBPs) detected from ticks and blood using 16S rRNA, gltA, and 18S rRNA query sequence similarities with GenBank subject sequences. [file 9958535.f1.docx]

**Table S1.** Numbers of blood samples obtained by district from small and large ruminants in Punjab, Pakistan.

| **Districts** | **Cow** | **Buffaloes** | **Goat** | **Sheep** |
| --- | --- | --- | --- | --- |
| Khushab | 32 | 32 | 9 | 13 |
| Bahawalnagar | 32 | 32 | 11 | 7 |
| Gujranwala | 32 | 32 | 6 | 7 |
| Kasur | 32 | 32 | 10 | 6 |
| Muzaffargarh | 32 | 32 | 13 | 8 |
| Sheikhupura | 32 | 32 | 8 | 5 |
| Vehari | 32 | 32 | 12 | 10 |
| Total | 224 | 224 | 69 | 56 |

**Table S2.** Primer details for molecular identification of ticks.

| **Organism** | **Primer name and sequence** | **Annealing temperature** | **Target gene** | **Product length (base pair)** | **References** |
| --- | --- | --- | --- | --- | --- |
| Ticks | Forward: 16S + 1 (5′-CTGCTCAATGATTTTTTAAATTGCTGTGG-3′)  Reverse: 16S-1 (5′-CCGGTCTGAACTCAGATCAAGT-3′) | 50°C° | 16S rRNA | 460 | (41) |
|  | Forward: LCO1490 (5′- GGTCAACAAATCATAAAGATATTGG-3′)  Reverse: HCO2198 (5′-TAAACTTCAGGGTGACCAAAAAATCA-3′) | 50°C° | COX1 | 710 | (42) |

**Table S3**. Primer details for molecular identification of pathogens.

| **Organism** | **Primer name and sequence** | **Annealing temperature** | **Target gene** | **Product length** | **References** |
| --- | --- | --- | --- | --- | --- |
| *Theileria* spp. and *Babesia* spp. | RLBF2: GACACAGGGAGGTAGTGACAAG  RLBR2: CTAAGAATTTCACCT CTGACA GT | 54°C for 30S | 18S rRNA | 460-540 bp | (44, 45) |
| *Ehrlichia* spp. and *Anaplasma* spp. | EHR16SF: GGTACCYACAGAAGAAGTCC  EHR16SR:  TAGCACTCATCGTTTACAGC | 54°C for 30 second | 16S rRNA | 345 bp | (46) |
| *Rickettsia*  spp. | Rick-F1: GAACGCTATCGG TATGCT TAACACA  Rick-R2 : CATCACTCACTCGGTATT GCTGGA | 60°C | 16S rRNA | 364 bp | (47) |
|  | CS78: GCAAGTATCGGTGAGGATGTAAT  CS238:  GCTTCCTAAAATTCAATAAATCAGGAT | 55°C | *gltA* | 401 bp | (46) |
| *Borrelia*  spp. | Forward: B16S_FL, GAC TCG TCA AGA CTG ACG CTA AGT C  Reverse: r B16S_R, GCA CAC TTA ACA CGT TAG CTT CGG TAC TAAC | 58°C | 16S rRNA | 131 bp | (48) |

**Table S4**. Species and numbers of of ticks collected by district and host, along with their accession numbers.

| Districts | Host | Tick species | Adult Male | Adult Female | Nymph | 16S | Cox1 |
| --- | --- | --- | --- | --- | --- | --- | --- |
| Vehari | Cattle | *R. microplus* | 07 | 21 | 00 | ON679614 | OQ380655 |
|  | Sheep | *R. microplus* | 13 | 17 | 01 | ON679619 |  |
|  | Buffalo | *H. anatolicum* | 18 | 13 | 03 | ON679620 | OQ380647 |
|  | Goat | *Rh. haemaphysaloides* | 00 | 03 | 01 | OQ379313 | OQ380646 |
| Sheikhupura | Cattle | *R. microplus* | 13 | 18 | 00 | ON679617 | OQ380657 |
|  |  | *H. anatolicum* | 09 | 17 | 00 | ON679623 | OQ380650 |
|  | Sheep | *H. anatolicum* | 00 | 08 | 01 | ON679625 | OQ380652 |
|  |  | *R. microplus* | 07 | 00 | 02 | OQ379305 | OQ380638 |
|  | Goat | *H. sulcata* | 04 | 00 | 00 | OQ379306 | OQ380639 |
|  |  | *R. turanicus* | 00 | 06 | 00 | OQ379309 | OQ380642 |
|  |  | *Rh. haemaphysaloides* | 01 | 04 | 00 | OQ379310 | OQ380643 |
|  | Buffalo | *R. turanicus* | 12 | 08 | 00 | ON679633 |  |
| Gujranwala | Cattle | *R. microplus* | 12 | 01 | 00 | ON679616 | OQ380656 |
|  | Sheep | *R. microplus* | 08 | 02 | 01 | OQ379301 | OQ380634 |
|  |  | *H. anatolicum* | 01 | 00 | 00 | OQ379302 | OQ380635 |
|  |  | *R. turanicus* |  |  |  |  |  |
|  | Buffalo | *H. anatolicum* | 05 | 16 | 0 | ON679621 | OQ380648 |
| Kasur | Goat  Goat | *R. microplus* | 19 | 02 | 01 | ON679618 | OQ380658 |
|  |  | *H. anatolicum* | 01 | 11 | 00 | ON679624 | OQ380651 |
|  |  | *Ha. sulcata* | 00 | 01 | 02 | OQ379304 | OQ380637 |
|  |  | *Ha. bispinosa* | 05 | 00 | 00 | ON679628 |  |
|  | Cattle | *Ha. bispinosa* | 04 | 02 | 00 | ON679626 |  |
|  |  | *H. scupense* | 03 | 01 | 00 | ON679629 |  |
|  | Buffalo | *R. decoloratus* | 11 | 05 | 00 | ON679631 |  |
|  |  | *Ha. bispinosa* | 00 | 18 | 01 | ON679627 |  |
|  | Sheep | *H. sulcata* | 00 | 03 | 00 | OQ379300 | OQ380633 |
| Bahawalnagar | Cattle | *H. anatolicum* | 12 | 02 | 01 | ON679622 | OQ380649 |
|  | Sheep | *A.variegatum* | 0 | 2 | 0 | ---- | ---- |
| Khushab | Cattle | *H. scupense* | 01 | 07 | 01 | ON679630 |  |
|  |  | *A.variegatum* | 0 | 2 | 0 | ----- | ----- |
|  |  | *H. anatolicum* | 00 | 12 | 00 | ------- | OQ380654 |
|  | Goat | *R. microplus* | 11 | 06 | 00 | OQ379312 | OQ380645 |
|  | Buffalo | *R. microplus* | 1 | 08 | 01 | ------- | OQ380661 |
| Muzaffargarh | Cattle | *R. decoloratus* | 15 | 09 | 00 | ON679632 |  |
|  | Buffalo | *H. scupense* | 04 | 02 | 00 | ------ | OQ380664 |
|  | Goat | *H. anatolicum* | 05 | 02 | 00 | ON679633 | OQ380644 |
|  |  | *A. variegatum* | 0 | 2 | 0 | ---- | ---- |
|  | Sheep | *H. anatolicum* | 07 | 07 | 01 | ------- | OQ380653 |
|  |  | *R. microplus* | 08 | 03 | 01 | ------- | OQ380659 |
| Total |  |  | 219 | 239 | 18 |  |  |

**Table S5**. Tick *16S rRNA* query sequences similarities with GenBank subject sequences.

| **Species** | **Accession ID**  **Current study** | **GenBank/Reference**  **Accession number** | **Similarity** | **Country** |
| --- | --- | --- | --- | --- |
| District Vehari, Pakistan | | | | |
| *R. microplus* | ON679614 | MN726558 | 100 | Pakistan |
| *R. microplus* | ON679619 | MG811555 | 99.97 | India |
| *H. anatolicum* | ON679620 | GU222462 | 99. 87 | India |
| R. haemaphysaloides | OQ379313 | MZ436882 | 99.79 | Pakistan |
| District Sheikhpura, Pakistan | | | | |
| *R. microplus* | ON679617 | KU664521 | 99.78 | China |
| *H. anatolicum* | ON679623 | MN326510 | 99.67 | India |
| *H. anatolicum* | ON679625 | MN326510 | 99.86 | Pakistan |
| *R. microplus* | 0Q379305 | KU895511 | 99.67 | India |
| *H. sulcata* | OQ379306 | KU130469 | 99.99 | Pakistan |
| *R. turanicus* | OQ379309 | MT79954 | 99.89 | Pakistan |
| R. haemaphysaloides | OQ379310 | MZ436882 | 99.79 | Pakistan |
| *R. turanicus* | ON679633 | OP352777 | 99.67 | Germany |
| District Gujrawala, Pakistan | | | | |
| *R. microplus* | ON679616 | MZ436881 | 99.45 | Pakistan |
| *R. microplus* | OQ379301 | MK455916 | 99.87 | Pakistan |
| *H. anatolicum* | OQ379302 | KY583069 | 99.67 | China |
| *H. anatolicum* | ON679621 | KR809584 | 99.67 | Pakistan |
| District Kasur, Pakistan | | | | |
| *R. microplus* | ON679618 | MT79946 | 99.87 | Pakistan |
| *H. anatolicum* | ON679624 | MN72655 | 99.78 | Pakistan |
| *H. sulcata* | OQ379304 | KR809584 | 99.67 | Pakistan |
| *H. bispinosa* | ON679628 | MT509435 | 99.67 | China |
| *H. bispinosa* | ON679626 | MF002560 | 99.87 | China |
| *H. scupense* | ON679629 | KY111474 | 99.67 | Afghanistan |
| *R. decoloratus* | ON679631 | MF002558 | 99.87 | China |
| *H. bispinosa* | ON679627 | MF002560 | 99.87 | China |
| *H. sulcata* | OQ379300 | MT79995 | 99.67 | Pakistan |
| District Bahawalnagar, Pakistan | | | | |
| *H. anatolicum* | ON679622 | KR890554 | 99.87 | Pakistan |
| District Khushab, Pakistan | | | | |
| *H. scupense* | ON679630 | KU130469 | 99.99 | Pakistan |
| *R. microplus* | OQ379312 | MT79954 | 99.89 | Pakistan |
| District MuzaffargarhR, Pakistan | | | | |
| *R. decoloratus* | ON679632 | OP352777 | 99.67 | Germany |
| *H. anatolicum* | ON679633 | KU130469 | 99.99 | Pakistan |

**Table S6**. Tick *Cox1* query sequences similarities with GenBank subject sequences.

| **Species** | **Accession ID**  **Current study** | **GenBank/Reference**  **Accession number** | **Similarity** | **Country** |
| --- | --- | --- | --- | --- |
| District Vehari, Pakistan | | | | |
| *R. microplus* | OQ380655 | KY606227 | 100 | Pakistan |
| *H. anatolicum* | OQ380647 | KU880577 | 99.97 | China |
| R. haemphysaloides | OQ380646 | OP050242 | 99. 87 | China |
| District Sheikhpura, Pakistan | | | | |
| *R. microplus* | OQ380657 | KT820180 | 99.78 | Iran |
| *H. anatolicum* | OQ380650 | MN728993 | 99.67 | Pakistan |
| *H. anatolicum* | OQ380652 | MT800319 | 99.86 | Pakistan |
| *R. microplus* | OQ380638 | MT800321 | 99.67 | Pakistan |
| *H. sulcata* | OQ380639 | KU130634 | 99.99 | Pakistan |
| *R. turanicus* | OQ380642 | KU130579 | 99.89 | Pakistan |
| Rh. haemphysaloides | OQ380643 | KU130649 | 99.79 | Pakistan |
| District Gujrawala, Pakistan | | | | |
| *R. microplus* | OQ380656 | MT108550 | 99.67 | Tunisia |
| *R. microplus* | OQ380634 | MT532304 | 99.45 | Iran |
| *H. anatolicum* | OQ380635 | OK623472 | 99.87 | Pakistan |
| District Kasur, Pakistan | | | | |
| *R. microplus* | OQ380658 | MN8553166 | 99.87 | China |
| *H. anatolicum* | OQ380651 | MH208696 | 99.78 | China |
| *H. sulcata* | OQ380637 | MG459961 | 99.67 | Bangladesh |
| *H. sulcata* | OQ380633 | MG459963 | 99.67 | Pakistan |
| District Bahawalnagar, Pakistan | | | | |
| *H. anatolicum* | OQ380649 |  | 99.67 | China |
| District Khushab, Pakistan | | | | |
| *H. anatolicum* | OQ380654 | MT80321 | 99.87 | China |
| *R. microplus* | OQ380645 | MT820180 | 99.67 | Iran |
| *R. microplus* | OQ380661 | MN8553166 | 99.87 | China |
| District Muzaffargarh, Pakistan | | | | |
| *H. scupense* | Oq380664 | KY606227 | 99.67 | Pakistan |
| *H. anatolicum* | Oq380659 | KU130634 | 99.87 | Pakistan |

**Table S7**. . Microorganisms detected through real-time microfluidic PCR in the individual tick species, collected from bovines in the districts of Kasur and Sheikhupura.

| **Bovine ID#** | **An_ma msp1** | **An_ov msp4** | **Anaplasma_spp_16S** | **Ehrlichia_spp_16S** | **Ri_aes ITS** | **Rickettsia_spp_gltA** | **Apicomplexa_18S** | **Theileria_spp_18S** | **Scientific Name** |
| --- | --- | --- | --- | --- | --- | --- | --- | --- | --- |
| **Kasur** | | | | | | | | | |
| 1* | + | - | + | - | - | - |  |  | [uncultured *Anaplasma sp. Anaplasma marginale Anaplasma centrale*](https://www.ncbi.nlm.nih.gov/Taxonomy/Browser/wwwtax.cgi?id=319051) |
| 2 | + | - | + | - | - | - |  | - |  |
| 3 | + | - | + | - | - | - |  |  |  |
| 4 | - | - | - | + | - | - |  | - | [uncultured *Anaplasma sp. Anaplasma marginale Anaplasma centrale*](https://www.ncbi.nlm.nih.gov/Taxonomy/Browser/wwwtax.cgi?id=319051) |
| 5 | _+ | - | + | - | - | - |  |  |  |
| 6 | + | - | + | - | - | - |  |  |  |
| 7 | - | + | + | - | - | - | - | - |  |
| 8 | + | - | + | - | - | - |  |  |  |
| 9 | + | - | + | - | - |  | - | - | [uncultured *Anaplasma sp. Anaplasma marginale Anaplasma centrale Anaplasma ovis*](https://www.ncbi.nlm.nih.gov/Taxonomy/Browser/wwwtax.cgi?id=319051) |
| 10 | - | - | - | - | + | + |  | - | [*Rickettsia sp. Rickettsia sp. TwKM01 Candidatus Rickettsia shennongii*](https://www.ncbi.nlm.nih.gov/Taxonomy/Browser/wwwtax.cgi?id=789) |
| 11 | - | + | + | - | - |  |  | - |  |
| 12 | - | - | - | - | - | - | + | + |  |
| 13 | - | - | - | - | - | - | + | + |  |
| 14 | + | - | + | - | - | - |  | - | [uncultured *Anaplasma sp. Anaplasma marginale Anaplasma centrale Anaplasma ovis*](https://www.ncbi.nlm.nih.gov/Taxonomy/Browser/wwwtax.cgi?id=319051) |
| **Sheikhupura** | | | | | | | | | |
| 15 |  | + | + | - | - | - |  |  |  |
| 16 | + | - | + | - | - | - | - | - |  |
| 17 | + | - | + | - | - | - | + | + | [*Theileria annulata*](https://www.ncbi.nlm.nih.gov/Taxonomy/Browser/wwwtax.cgi?id=5874) |
| 18* | - | + | + | - | - | - |  | - | [*Anaplasma ovis*](https://www.ncbi.nlm.nih.gov/Taxonomy/Browser/wwwtax.cgi?id=142058) |
| 19 | + | - | + | - | - | - | + | + |  |
| 20 | + | - | + | - | - | - | - | - |  |
| 21 | + | - | + | - | - | - |  |  |  |
| 22 | + | - | + | - | - | - |  | - |  |
| 23 | + | - | + | - | - | - |  |  |  |
| 24 | - | + | + | - | - | - | - | - |  |

**Table S8**. Microorganisms detected through real-time microfluidic PCR in the blood samples of bovine populations in the study districts of Kasur and Sheikhupura.

| **Bovine ID#** | **An_ma msp1** | **An_ov msp4** | **Anaplasma_spp_16S** | **Ehrlichia_spp_16S** | **Ri_aes ITS** | **Rickettsia_spp_gltA** | **Apicomplexa_18S** | **Theileria_spp_18S** | **Scientific Name** |
| --- | --- | --- | --- | --- | --- | --- | --- | --- | --- |
| **Kasur** | | | | | | | | | |
| 1* | + | - | + | - | - | - | + | + | [*uncultured Anaplasma sp. Anaplasma marginale Anaplasma centrale*](https://www.ncbi.nlm.nih.gov/Taxonomy/Browser/wwwtax.cgi?id=319051) |
| 2 | - | + | + | - | - | - | + | + | [*Theileria lestoquardi*](https://www.ncbi.nlm.nih.gov/Taxonomy/Browser/wwwtax.cgi?id=77054) |
| 3 | - | - |  | - | - |  | + | + |  |
| 4 | - | + | + | - | - | - | - | - |  |
| 5 | - | + | + | - | - | - |  | - |  |
| 6 | - | + | + | - | - | - |  | - |  |
| 7 | - | - |  | - | - | - | + | - | [*Babesia bigemina*](https://www.ncbi.nlm.nih.gov/Taxonomy/Browser/wwwtax.cgi?id=5866) |
| 8 | + | - | + | - | - | - | + | + | [*Theileria annulata*](https://www.ncbi.nlm.nih.gov/Taxonomy/Browser/wwwtax.cgi?id=5874) |
| 9 | + | - | + | - | - | - |  | - |  |
| 10 | + | - | + | - | - | - | + | + | [*Theileria annulata*](https://www.ncbi.nlm.nih.gov/Taxonomy/Browser/wwwtax.cgi?id=5874) |
| 11 | + | - | + | - | - | - | - | - | [uncultured *Anaplasma sp. Anaplasma marginale Anaplasma centrale*](https://www.ncbi.nlm.nih.gov/Taxonomy/Browser/wwwtax.cgi?id=319051) |
| 12 | + | - | + | - | - | - |  |  |  |
| 13 | + | - | + | - | - | - |  |  |  |
| 14 | - | - | + | - | - | - | + | + |  |
| 15 | - | + | + | - | - | - | - | - | [*Anaplasma ovis*](https://www.ncbi.nlm.nih.gov/Taxonomy/Browser/wwwtax.cgi?id=142058) |
| **Sheikhupura** | | | | | | | | | |
| 16 | - | + | + | - | - | - | - | - |  |
| 17 | - | - | - | - | - | - | + | + |  |
| 18* | - | + | + | - | - |  |  |  | [*Anaplasma ovis*](https://www.ncbi.nlm.nih.gov/Taxonomy/Browser/wwwtax.cgi?id=142058) |
| 19 | - | + | + | - | - | - | - | - |  |
| 20 | + | - | + | - | - | - |  | - |  |
| 21 | - | + | + | - | - | - | - | - |  |
| 22 | - | - | + | - | - | - | + | + | [*Anaplasma platys* uncultured *Anaplasma sp. Candidatus Anaplasma cinensis Candidatus Anaplasma camelii Anaplasma sp. AspGDr3*](https://www.ncbi.nlm.nih.gov/Taxonomy/Browser/wwwtax.cgi?id=949) |
| 23 | - | + | + | - | - | - |  | - |  |
| 24 | + | - | + | - | - | - |  |  |  |
| 25 | - | - | - | - | - |  | + |  |  |
| 26 | - | + | + | - | - | - | - |  |  |
| 27 |  | + | + | - | - | - |  | - |  |
| 28 | - | - | - | - | - | - | + | + |  |
| 29 | + | - | + | - | - | - | + | + |  |
| 30 |  | + | + | - | - | - |  |  |  |
| 31 | + | - | + | - | - |  |  |  |  |
| 32 | + | - | + | - | - | - |  |  |  |
| 33 | + | - | + | - | - | - |  |  |  |
| 34 | - | + | + | - | - | - | - | - | [*Anaplasma ovis*](https://www.ncbi.nlm.nih.gov/Taxonomy/Browser/wwwtax.cgi?id=142058) |
| 35 | + | - | + | - | - | - |  |  |  |
| 36 | + | - | + | - | - | - |  |  |  |
| 37 | - | - | - | - | - | - | + | + |  |
| 38 | - | + | + | - | - | - | - | - |  |
| 39 | + | - | + | - | - | - |  | - |  |
| 40 | - | + | + | - | - | - | - | - | [*Anaplasma ovis*](https://www.ncbi.nlm.nih.gov/Taxonomy/Browser/wwwtax.cgi?id=142058) |
| 41 | + | - | + | - | - | - | + | + |  |
| 42 | - | - | - | - | - | - | + | + |  |
| 43 | 1 | - | + | - | - | - | + | + |  |
| 44 | - | + | + | - | - | - |  |  |  |

*Single infection and co-infection detection from blood and tick samples collected from same animal

**Table S9**. Tick-borne pathogens (TBPs) detected from ticks and blood by using 16S rRNA, gltA and 18S rRNA query sequence similarities with GenBank subject sequences.

| **Species** | **Accession ID**  **Current study** | **GenBank/Reference**  **Accession number** | **Similarity** | **Country** |
| --- | --- | --- | --- | --- |
| *Rickettsia* species 16S rRNA | | | | |
| *Rickettsia sp.* | OQ533599 | KF318168 | 99.67 | Thailand |
| *Rickettsia sp.* | OQ533600 | MZ851175 | 99.75 | India |
| *Rickettsia sp.* | OQ533601 | MZ851176 | 99.54 | India |
| *R. massilliae* | OQ533696 | ON076427 | 99.46 | Egypt |
| *R. massilliae* | OQ533697 | KY575386 | 99.98 | Spain |
| *R. massilliae* | OQ533698 | MZ851177 | 99.76 | India |
| *R. hoogstraalii* | OQ581856 | KY575384 | 99.87 | Spain |
| *Rickettsia* species gltA | | | | |
| *R. massilliae* | OQ59935 | MW430407 | 99.67 | Kazakhstan |
| *R. massilliae* | OQ59936 | MW422252 | 99.68 | Kazakhstan |
| *R. slovaca* | OQ59939 | MN388796 | 99.65 | China |
| *R. slovaca* | OQ59940 | KY418024 | 99.45 | Itlay |
| *R. slovaca* | OQ59941 | KU948238 | 99.45 | Malaysia |
| *R. hoogstraalii* | OQ59942 | MW802693 | 99.87 | China |
| *Anaplasma* species | | | | |
| *A. bovis* | OQ533602 | MK680807 | 100 | Pakistan |
| *A. marginale* | OQ533603 | MK680804 | 99.87 | Pakistan |
| *Anaplasma spp.* | OQ533604 | MZ558066 | 99.45 | India |
| A. capra | OQ547105 | MK991954 | 99.45 | China |
| *A. capra* | OQ547106 | MH255930 | 99.70 | China |
| *Ehrlichia* species | | | | |
| *Ehrlichia sp.* | OQ545727 | M250197 | 99.86 | Pakistan |
| *Theileria* and *Babesia* Spcies | | | | |
| *T. orientalis* | OQ550152 | MK8338120 | 100 | Pakistan |
| *T. orientalis* | OQ550154 | KT73149 | 99.97 | India |
| *T. orientalis* | OQ550155 | OQ550168 | 99. 87 | Pakistan |
| *T. annulata* | OQ550156 | MN960099 | 100 | Iran |
| *T. annulata* | OQ550157 | MG585372 | 99.75 | Pakistan |
| *T. annulata* | OQ550158 | MT3182210 | 99.54 | Pakistan |
| *T. annulata* | OQ550159 | MK918607 | 99.46 | Turkey |
| *T. annulata* | OQ550160 | FJ603460 | 99.98 | China |
| *T. annulata* | OQ550161 | MH208641 | 99.76 | China |
| *T. annulata* | OQ550162 | MK8338120 | 99.87 | Pakistan |
| *T. annulata* | OQ550160 | EU622911 | 100 | France |
| *T. annulata* | OQ550163 | MT498783 | 99.87 | Pakistan |
| *T. ovis* | OQ550164 | MF287950 | 99.45 | India |
| *T. ovis* | OQ550165 | MT318210 | 99.45 | Pakistan |
| *T. ovis* | OQ550166 | MG585379 | 99.70 | Pakistan |
| *Theileria sp.* | OQ550167 | MG208641 | 99.87 | China |
| *Theileria sp.* | OQ550168 | MG585379 | 99.45 | Pakistan |
| *B. bovis* | OQ550169 | L190178 | 99.45 | South Africa |
| *B. bigemina* | OQ550170 | EF458206 | 99.70 | Germany |


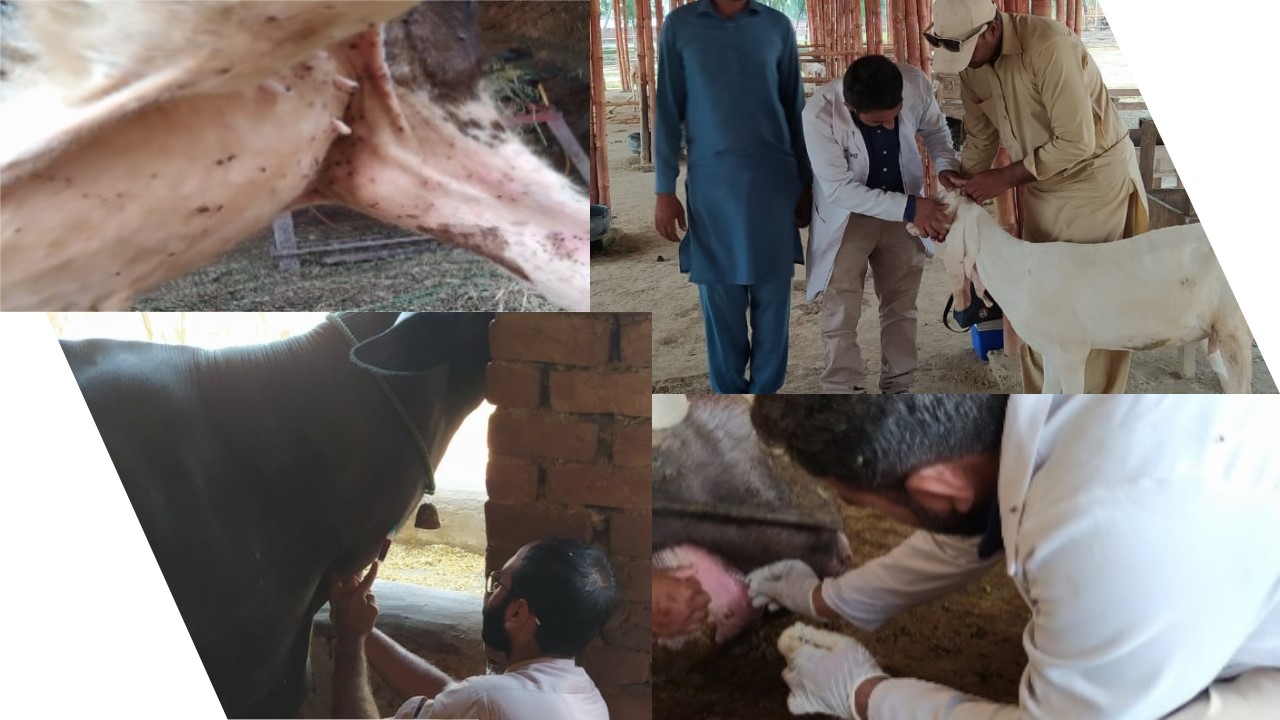


**Figure S1** Blood collection details from small and large ruminants in Punjab, Pakistan.
